# Efficient Sn Recovery from SnO_2_ by Alkane (C_*x*_H_*y*=2*x*+2_, 0 ≤ *x* ≤ 4) Reduction

**DOI:** 10.1038/s41598-019-53389-7

**Published:** 2019-11-13

**Authors:** Hyesung An, Mi Yoo, Hyunwoo Ha, Hyuk Choi, Eunji Kang, Hyun You Kim

**Affiliations:** Department of Materials Science and Engineering, Chungnam National University, 99 Daehak-ro, Yuseong-gu, Daejeon, 34134 Republic of Korea

**Keywords:** Metals and alloys, Design, synthesis and processing, Theory and computation

## Abstract

We study the mechanism of alkane reduction of SnO_2_ for efficient low-temperature recovery of Sn from SnO_2_. Based on thermodynamic simulation results, we comparatively analyze the reduction behavior and the efficiency of SnO_2_ reduction by H_2_ and alkanes (C_*x*_H_y=2*x+2*_, 0 ≤ *x* ≤ 4). We found that alkanes (*n*·C_*x*_H_*y*_) with the higher *nx* generally complete the reduction of SnO_2_ (T_100_) at the lower temperature. Moreover, the T_100_ of the SnO_2_ reduction by alkanes (*n*·C_*x*_H_*y*_) was decreased from the T_100_ of pure hydrogen with the same amount of hydrogen atoms (*n*·H_*y*_). We found that the concentration of a gas phase product mixture, the amount of the produced solid carbon, and the T_100_ complementary vary as a function of the *nx* and *ny*, the total amount of carbon and hydrogen atoms in the reducing gas phase molecules. Our results demonstrate a viability of the low temperature reduction method of SnO_2_ by alkanes for efficient recovery of Sn from SnO_2_, which can be applied for Sn recovery from Sn containing industrial wastes or Sn ores with economic value added that is held by the co-produced H_2_.

## Introduction

Recovering (extracting) valuable metallic elements from industrial wastes is technically important for the efficient recycle of earth unabundant resources. However, the current dry-smelting, hydro-smelting, or combined smelting-electrolytic refining technologies, which are commonly applied for extraction of high purity metals from ores or used oxidized scraps, are not environmentally friendly^1–9^. Because the high quality minable ores deplete first, designing environmentally friendly techniques are necessary to set up ecofriendly recovery processes of used metals.

Although Sn (Tin) is relatively earth unabundant among the industrially demanded metals, Sn and Sn oxides play a key role in several electronic devices and products such as sensor^10,11^, Pb-free solder^12,13^, or transparent electrode^14–16^. Despite of the high LME (London Metal Exchange) market price ($16,450.00/metric ton as of October 4, 2019) of Sn^17^, which is more than 3 and 1.5 times expansive than Cu and Ni, respectively, 70% of the annually consumed Sn is not appropriately recycled^18^. Recovery of Sn from used SnO_2_ or oxidized metal scrap proceeds in a similar process with the ore smelting in the presence of a proper and strong reducer, usually cokes. However, a poor solid-solid contact between SnO_x_ and cokes and a high operation temperature lowers the overall efficiency^19^.

In our previous report^20,21^, we have designed a methane reduction (MR) method, an ecofriendly and simple versatile process of Sn recovery from SnO_x_ containing industrial wastes, which also can be potentially applied for Sn ore smelting. A direct facile contact between gas phase methane and SnO_*x*_ improves the efficiency of the reaction. Moreover, multiple reductants provided by methane (hydrogen and carbon) sequentially reduce SnO_x_, producing H_2_O, H_2_, CO, or CO_2_ depending on the reduction conditions^21^. The geometry of a SnO_2_ bound methane inhibits the initial participation of the carbon of methane to the reduction process^21^. Rather, the released hydrogen atoms from methane attribute to the initial reduction power of methane^21^. The extended release of two kinds of reducing agents from methane assures the versatility of the MR of SnO_x_ and the increased economic efficiency^21^. Another interesting finding was that the H_2_/CO ratio in the produced gas varies as a function of the CH_4_/SnO_2_ ratio. We found that the H_2_/CO ratio increases if the MR of SnO_x_ proceeds under oxygen depleted conditions because the late released more oxophilic carbon takes up oxygen atoms from SnO_x_ and gas phase H_2_O^21^.

Unique chemistry between Sn and CH_4_ has reported in a recent study by the Metiu and McFarland groups^22^. They found that molten Sn and other transition metals can directly dissociate CH_4_ into solid C and H_2_ and suggested that such direct H_2_ production from CH_4_ without CO_2_ formation as an advanced H_2_ production method from hydrocarbons^22^. Based on our previous findings on the CH_4_ reduction of SnO_2_^21^, we have reached to a hypothesis that use of alkanes with more carbon and hydrogen contents per mole (C_*x*_H_*y*=2*x*+2_, 0 ≤ *x* ≤ 4) would accelerate the SnO_2_ reduction and also lower the reaction temperature. Moreover, if molten Sn, which is produced upon SnO_2_ reduction by alkanes, assists dissociation of alkanes into carbon and hydrogen, the SnO_2_ reduction would occur under the stronger reduction atmosphere so that the overall SnO_2_ reduction will be greatly accelerated.

In this letter, we use a combined study of thermodynamic simulations and density functional theory (DFT) calculations to study the effect of the C/H_2_ ratio in the reducing gas on the efficiency of the reduction of SnO_2_. To provide a fundamental insight into the mechanism of SnO_2_ reduction by alkanes with the higher carbon and hydrogen contents per mole and further clarify the reduction potential of the applied alkanes, we introduce commercially available alkanes (C_*x*_H_*y*=2*x*+2_, 0 ≤ *x* ≤ 4) as a reducing agent for SnO_2_ reduction. The efficiency of the alkane reduction of SnO_2_ is evaluated by the reduction complete temperature, T_100_, and compared with the T_100_ of mole-balanced pure hydrogen. We find that the T_100_ is an inverse exponential function of the amount of supplied reducing agent (H_2_ or alkane) and that the addition of carbon as a form of alkane significantly lowers the T_100_ from that of the H_2_ reduction of SnO_2_. Our findings predict that the operation temperature of the alkane reduction of SnO_2_ can be adjusted by controlling the composition and the *x*/*y* ratio of the reducing gas, suggesting an easy and industrially highly accessible recycling process of SnO_*x*_ containing industrial wastes.

## Results and Discussion

### H_2_ reduction of SnO_2_

**Figure 1:**
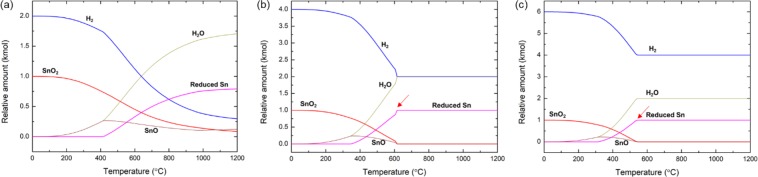
Theoretical prediction of the H_2_ reduction of SnO_2_. (**a**–**c**) Equilibrium concentration of the mixture of one mole of SnO_2_ and *n*∙CH_4_ (*n* = 2, 4, or 6) as a function of temperature. (**a**) H_2_/SnO_2_ = 2, (**b**) H_2_/SnO_2_ = 4, and (c) H_2_/SnO_2_ = 6. The red arrows in (**b**) nd (**c**) indicated the T_100_.

Figure 1 shows the equilibrium concentrations of the mixture of SnO_2_ and *n*∙H_2_ (*n* = 2, 4, or 6) at between 0 to 1200 °C. Obviously, H_2_ reduces SnO_2_ into Sn through a two-step process. In all cases, SnO_2_ was first transferred to SnO. SnO was formed at below the melting temperature of Sn (231.9 °C) and further transferred to metallic Sn upon temperature increase. Under the stoichiometric condition (H_2_/SnO_2_ = 2, 2 moles of H_2_ is required to reduce a mole of SnO_2_ to Sn and 2H_2_O), the reduction does not complete even at 1200 °C and SnO survives. The T_100_, at which SnO_2_ and SnO were completely reduced to Sn, was significantly decreased upon increase of the amount of supplied H_2_ up to 4 or 6 moles (H_2_/SnO_2_ = 4 or 6, respectively, Table 1). Because consistent two moles of H_2_ were used for SnO_2_ reduction to Sn, irrespective to the initial H_2_/SnO_2_ ratio, the decrease of the T_100_ is presumably due to the increased chemical potential of gas phase H_2_ upon increase in the H_2_/SnO_2_ ratio.

**Table 1:** T_100_ of *n*∙H_2_ reduction of SnO_2_ as a function of the amount of supplied H_2_, *n*.

Amount of supplied H_2_, *n*, (kmol)	2	4	6	8	9	10
T_100_ (°C)	n/a^a^	615	545	494	484	464

^a^Reduction was not completed up to 1200 °C.

### Alkane reduction of SnO_2_: methane and ethane

**Figure 2:**
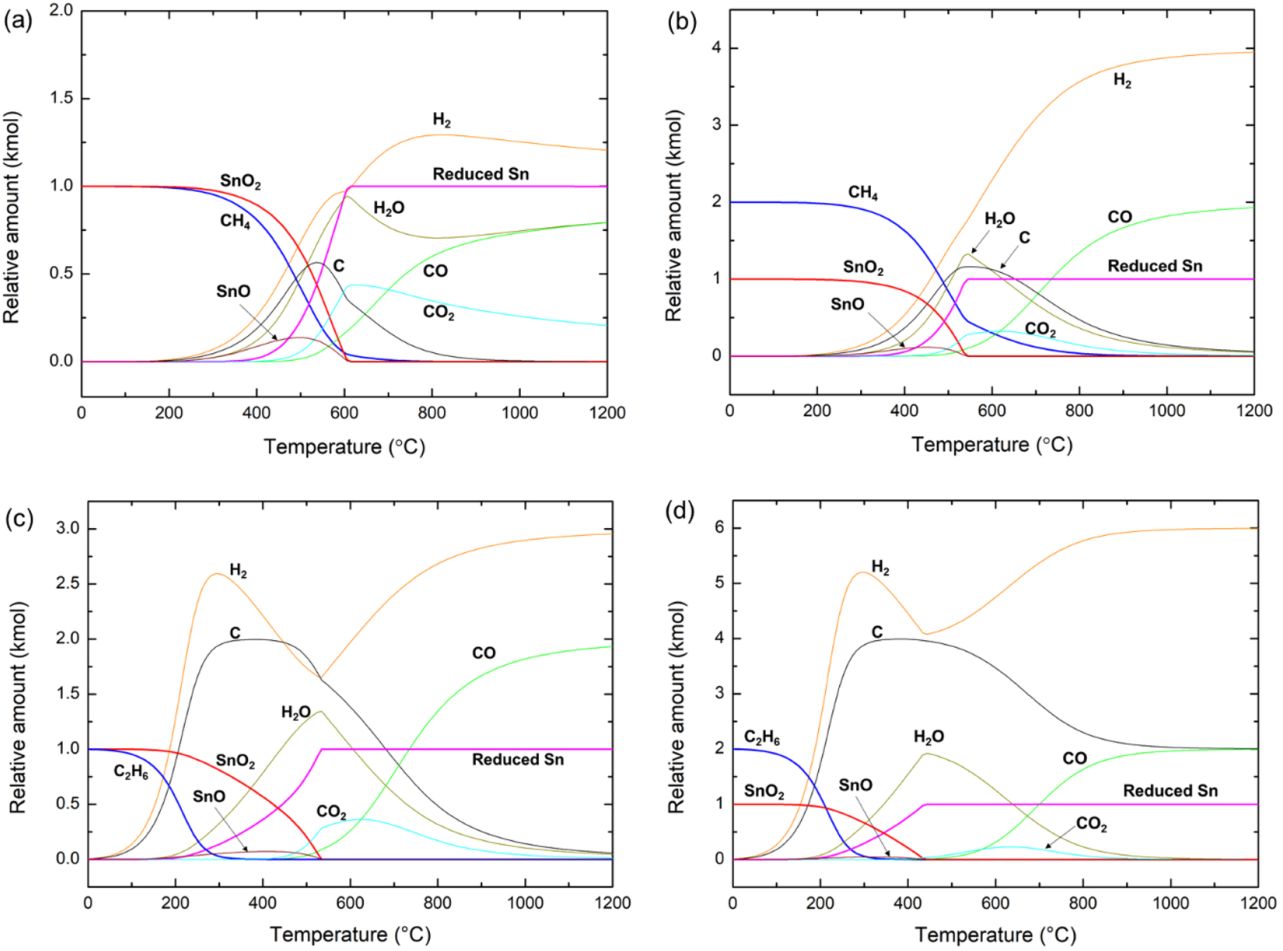
Temperature dependent evolution of equilibrium concentration of reactants and products during (**a**,**b**) CH_4_ or (**c**,**d**) C_2_H_6_ reduction of SnO_2_. (**a**) CH_4_ + SnO_2_, (**b**) 2CH_4_ + SnO_2_, (**c**) C_2_H_6_ + SnO_2_, and (**d**) 2C_2_H_6_ + SnO_2_.

Figure 2 shows the equilibrium concentrations of the mixture of SnO_2_ and *n*∙CH_4_ (Fig. 2a,b) or *n*∙C_2_H_6_ (Fig. 2c,d) (*n* = 2 or 4) at between 0 to 1200 °C. Like the cases of the H_2_ reduction of SnO_2_, the T_100_ is equal to the point at which the SnO and SnO_2_ are completely depleted. Because a mole of CH_4_ supplies total five units of reducing agents (one C and four H), a mole of SnO_2_ can be easily reduced to metallic Sn. The increase of H_2_, C, and H_2_O above 200 °C shows that CH_4_ was decomposed into C and H_2_ and the released H_2_ from CH_4_ initially reduces SnO_2_. The delayed increase of CO_2_ compared to the increase of C confirms that the reduction by C occurs at the higher temperature than the reduction by H_2_. In both cases (CH_4_/SnO_2_ = 2 or 4) the decrease of H_2_O, C, and CO_2_ is coupled with the increase of CO and H_2_, meaning that C takes up oxygen from SnO_2_ under C and H_2_ rich conditions. Despite the active role of hydrogen in the early stage of the reduction, carbon completes the reduction and hydrogen of CH_4_ was released as gas phase H_2_. The T_100_ of CH_4_ reduction of SnO_2_ also decreases response to the increase of the CH_4_/SnO_2_ ratio (Table 2).

**Table 2:** T_100_ of *n*∙C_*x*_H_*y*=2*x*+2_ (*x* = 1 or 2) reduction of SnO_2_ as a function of the amount of supplied alkanes, *n*.

*n*∙C_*x*_H_*y*=2*x*+2_ (*x* = 1 or 2), (*nx, ny*) (kmol)	1∙CH_4_ (1, 4)	2∙CH_4_ (2, 8)	1∙C_2_H_6_ (2, 6)	2∙C_2_H_6_ (4, 12)
T_100_ (°C)	615	545	534	444

The overall reduction process, initial active reduction of SnO_2_ by H_2_ and complete reduction by C, was consistently appeared in the C_2_H_6_ reduction of SnO_2_. C_2_H_6_ decomposes rapidly into C and H_2_ and the overall reduction occurs under highly reducible conditions. However, although the reduction occurs under C and H_2_ rich conditions, SnO was also appeared as an intermediate, showing that the reduction of SnO_2_ occurs through a two-step process. The rapidly increased H_2_ upon C_2_H_6_ decomposition gradually decreased as H_2_ was transformed to H_2_O. Like the case of CH_4_ reduction of SnO_2_, C takes up oxygen, being transformed to CO_2_ and eventually, to CO. Most of the H_2_ transformed to H_2_O was released upon CO formation. When the C_2_H_6_/SnO_2_ increases to 4, the excess C was remained as solid state carbon even after complete reduction of SnO_2_. The T_100_ values of C_2_H_6_ reduction of SnO_2_ were generally lower than the values of CH_4_ reduction of SnO_2_ (Table 2). The effect of the amount of C and H_2_ in reducing alkanes on the T_100_ will be discussed below.

### Alkane reduction of SnO_2_: propane and butane

**Figure 3:**
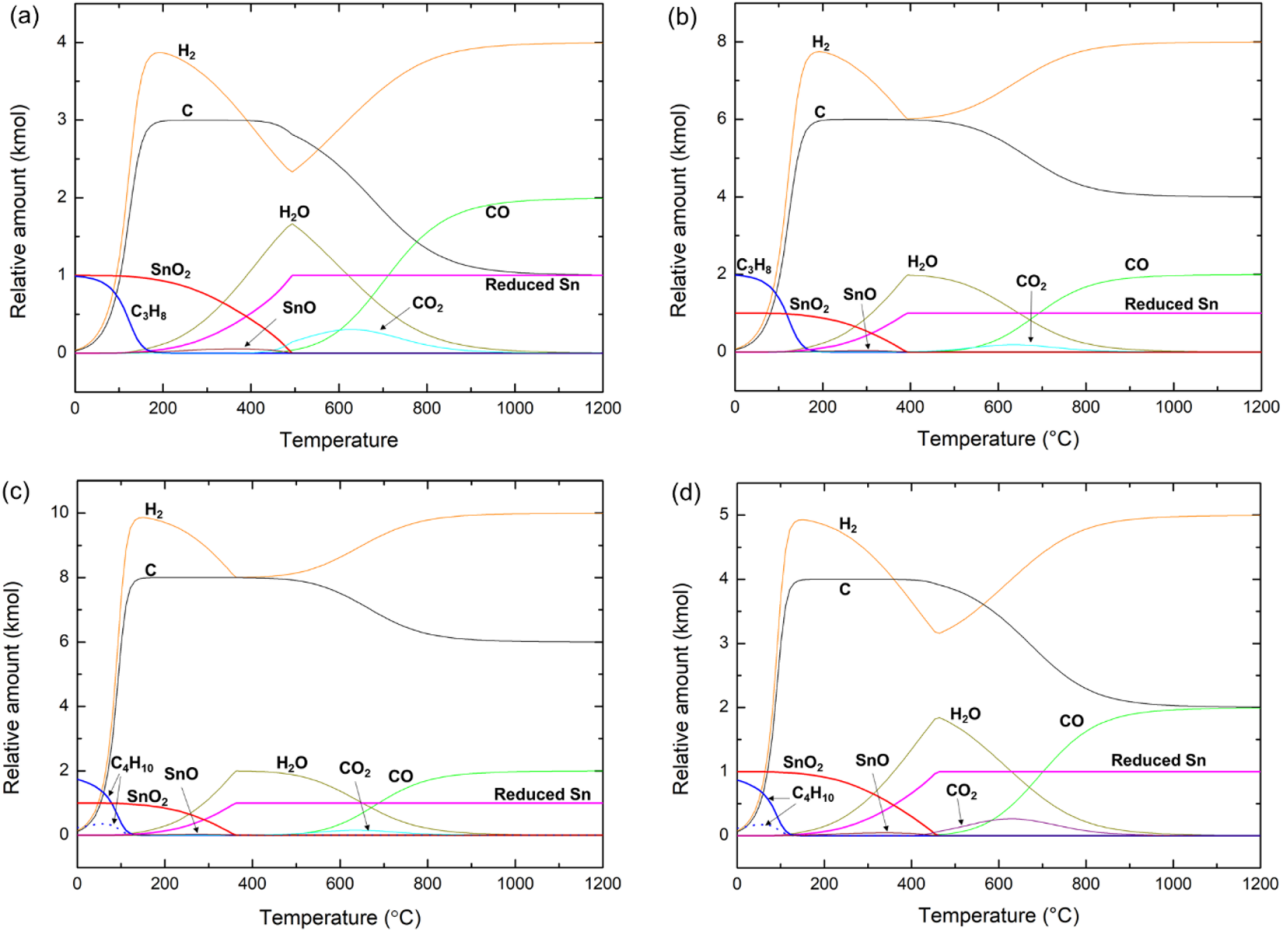
Temperature dependent evolution of equilibrium concentration of reactants and products during (**a**,**b**) C_3_H_8_ or (**c**,**d**) C_4_H_10_ reduction of SnO_2_. (**a**) C_3_H_8_ + SnO_2_, (**b**) 2C_3_H_8_ + SnO_2_, (**c**) C_4_H_10_ + SnO_2_, and (**d**) 2C_4_H_10_ + SnO_2_.

C_3_H_8_, propane, and C_4_H_10_, butane, are commercially widely available alkanes and a component of liquid petroleum gas. No meaningful changes in the reduction behavior was observed in the C_3_H_8_ and C_4_H_10_ reduction of SnO_2_ (Fig. 3). Decomposition of C_3_H_8_ and C_4_H_10_ caused a rapid increase of H_2_ and C in the initial state of the reduction. Gradual increase of H_2_O coupled with the increase of reduced Sn and SnO represents the initial reduction of SnO_2_ by H_2_ released from alkanes. Because the excess amount of H_2_ was supplied, even in the presence of solid state carbon, H_2_ takes up oxygen from SnO_2_. Subsequent reactions between H_2_O and solid state carbon produce CO_2_, CO, and H_2_. Eventually, all of the oxygen from SnO_2_ was converted to CO at high temperatures and the excess C and all of H_2_ from alkanes were released as is. Upon increase of the supplied C_3_H_8_ and C_4_H_10_, the T_100_ was also significantly reduced (Table 3). Interestingly, the formation of SnO was suppressed at C_3_H_8_/SnO_2_ = 2 and C_4_H_10_/SnO_2_ = 2. Direct reduction of SnO_2_ could become available under H_2_ rich conditions.

**Table 3:** T_100_ of *n*∙C_*x*_H_*y*=2*x*+2_ (*x* = 3 or 4) reduction of SnO_2_ as a function of the amount of supplied alkanes, *n*.

*n*∙C_*x*_H_*y*=2*x*+2_ (*x* = 3 or 4), (*nx, ny*) (kmol)	1∙C_3_H_8_ (3, 8)	2∙C_3_H_8_ (6, 16)	1∙C_4_H_10_ (4, 10)	2∙C_4_H_10_ (8, 20)
T_100_ (°C)	494	393	464	363

### Modelling the reduction trend in alkanes (*n·*C_x_H_y=2*x*+2_, 0 ≤ *x* ≤ 4)

**Figure 4:**
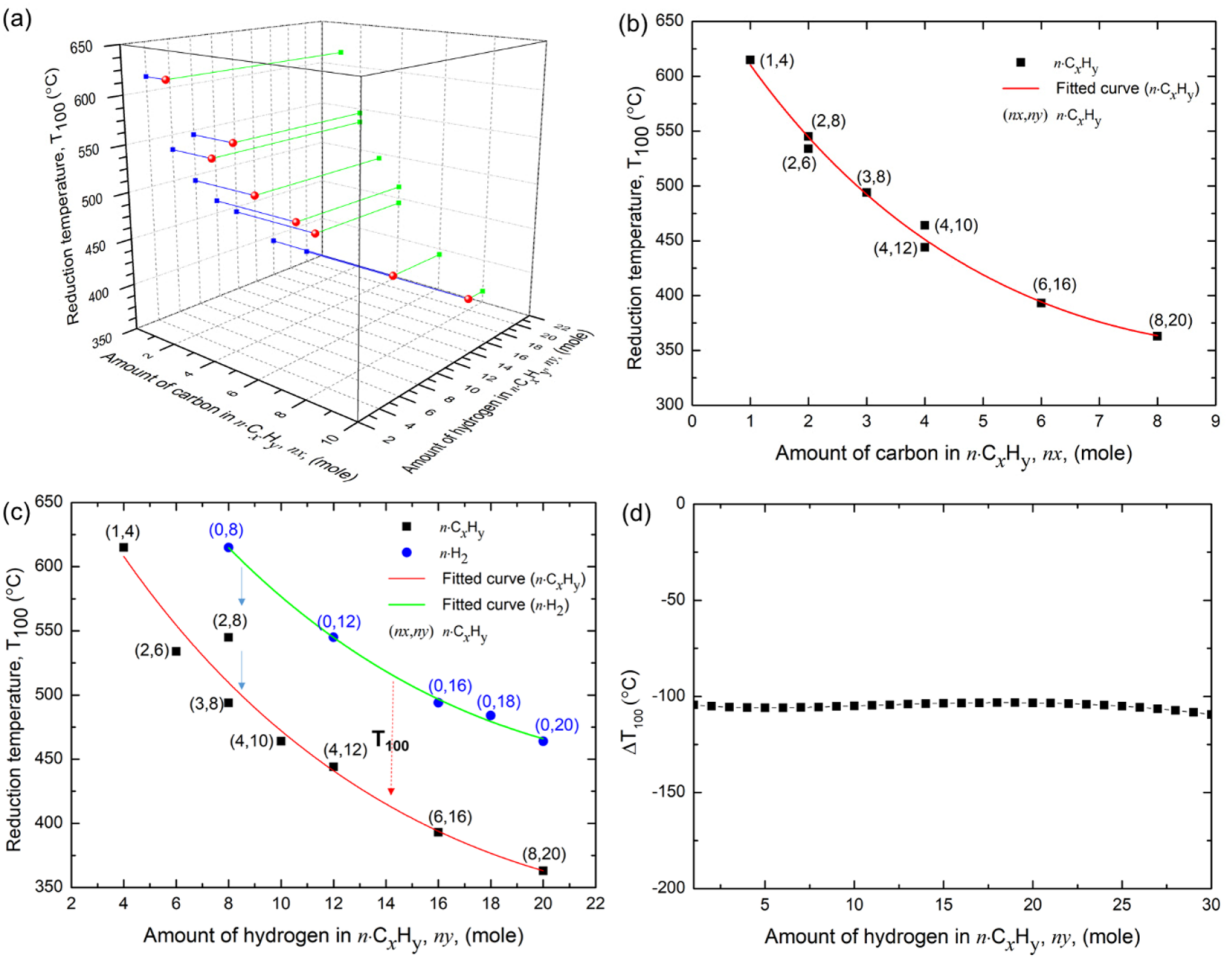
Effect of C on the T_100_ of H_2_ or alkane reduction of SnO_2_. (**a**) A 3-dimensional diagram of T_100_ of alkane reduction of SnO_2_ presented as a function of *nx* and *ny*. (**b**) T_100_ values of alkane reduction of SnO_2_ plotted as a function of *nx*. The pairs of numbers in the parentheses present the total units of C, *nx*, and H, *ny*, in alkanes. (**c**) T_100_ values of H_2_ or alkane reduction of SnO_2_ plotted as a function of *ny*. The pairs of numbers in the parentheses present the total units of C, *nx*, and H, *ny*, in reductants. Red and green lines present the fitted exponential functions of T_100_ as a function of *ny*. (**d**) ΔT_100_ values estimated from the two fitted exponential curves of T_100_.

The equilibrium concentration diagrams presented in Figs 1 and 2 show that the overall reduction process of SnO_2_ by H_2_ and alkanes (*n·*C_x_H_y=2*x*+2_, 0 ≤ *x* ≤ 4) does not differ a lot. Vigorous release of H_2_ at low temperatures from alkanes generates the similar reducing atmosphere with the reduction by pure H_2_. Addition of the released C from alkanes induces the gas phase conversion of H_2_O into H_2_. Moreover, as the alkane/SnO_2_ ratio increases from 1 to 2, the T_100_ decreases. The response of the T_100_ as a function of the total amount of supplied C, *nx*, and H, *ny*, is presented in Tables 2, 3 and Fig. 4a.

Considering that the T_100_ values are exponentially decreasing upon increase of *nx* and *ny* and that the addition of C affects to the T_100_, we presented the T_100_ values as a function of *nx* (Fig. 4b) or *ny* (Fig. 4c). Figure 4b shows that once the amount of C, *nx*, is given, the T_100_ just slightly varies upon change in *ny*, presenting the quite prominent and dominant effect of C on the T_100_ of SnO_2_ reduction by alkanes, as predicted by thermochemical data: the standard formation enthalpy of CO_2_, $\Delta {H}_{f}^{0}({{\rm{CO}}}_{2},{\rm{298.15}}\,K)=-{\rm{393.474}}\,\mathrm{kJ}/\mathrm{mol}$, is greater than that of water, $\Delta {H}_{f}^{0}({H}_{2}{\rm{O}},{\rm{298.15}}\,K)=-{\rm{285.830}}\,\mathrm{kJ}/\mathrm{mol}$^23^. Because single C atom can take over two O atoms from SnO_2_, whereas two H atoms are required to remove one O atom from SnO_2_, C of alkanes will naturally more aggressively reduce SnO_2_.

In Fig. 4c, to more intensively compare the effect of C on the T_100_ of SnO_2_, we presented a pair of dataset, the T_100_ values of H_2_ or alkane reduction of SnO_2_ as a function of *ny*. The control group data, the T_100_ values acquired from H_2_ reduction of SnO_2_, gradually decrease as a function of *ny*: 615 °C at H_2_/SnO_2_ = 8 and 464 °C at H_2_/SnO_2_ = 20. The filled square data points in Fig. 4c represent the T_100_ of SnO_2_ reduction by *n·*CH_4_, *n·*C_2_H_6_, *n·*C_3_H_8_, and *n·*C_4_H_10_. For the cases where two or more combinations of alkanes are available to match the total amount of supplied hydrogen, *ny*, we took the case with the higher *nx*. For example, we took the T_100_ from 2C_2_H_6_ (*nx* = 4) rather than that from 3CH_4_ (*nx* = 3), to compare with the T_100_ from 6H_2_ (*ny* = 12). The T_100_ values of alkane reduction of SnO_2_, fitted to an exponential function, show a significantly decrease in T_100_ (Fig. 4c). Replacing a reducing agent from 4 moles of H_2_ (*ny* = 8) to a mole of C_3_H_8_ (*nx* = 3, *ny* = 8) decreased the T_100_ of a mole of SnO_2_ from 615 °C to 494 °C (Fig. 4c). The fitted exponential curves of T_100_ as a function of *nx* or *ny* (solid lines in Fig. 4b,c) show that the T_100_ of the alkane or H_2_ reduction can be fit to simple exponential functions (refer to Tables 4 and 5 for fitting constants and R^2^-values).

**Table 4:** T_100_ of alkane reduction of SnO_2_ fitted to an exponential function of *nx*.

Reference function	T_100_ = *exp*[*a* + b (*nx*) + c(*nx*)^2^], (1 ≤ *nx* ≤ 10)			
Fitting parameters	a	b	c	Adjusted R^2^
*n*·C_*x*_H_*y*_ reduction of SnO_2_	6.5413	−0.1340	0.0067	0.9898

**Table 5:** The T_100_ of H_2_ or alkane reduction of SnO_2_ fitted to an exponential function of *ny*.

Reference function	T_100_ = *exp*[*a* + b (*ny*) + c(*ny*)^2^], (0 ≤ *ny* ≤ 20)			
Fitting parameters	a	b	c	Adjusted R^2^
H_2_ reduction of SnO_2_	6.7477	−0.0479	8.8363 × 10^−4^	0.9956
*n*·C_*x*_H_*y*_ reduction of SnO_2_	6.6350	−0.0629	0.0013	0.9834

Interestingly, we found that the ΔT_100_ (T_100_-alkane – T_100_-hydrogen), an indicator of the effect of carbons from alkanes on the reduction of SnO_2_ was −121 °C at *ny* = 3 and rapidly saturated to −101 °C at *ny* = 4 and beyond (Fig. 4c). The ΔT_100_ calculated from the two fitted exponential curves predicts the slightly fluctuating ΔT_100_ centered at −105 °C (Fig. 4d). Because the ΔT_100_ was estimated comparing the (0, *ny*) and (*nx*, *ny*) data points with the maximum *nx* value, it naturally represents the maximum effect of C addition to the T_100_ of SnO_2_ reduction. The overall increase of *nx* and *ny* is beneficial for SnO_2_ reduction because the lower T_100_ assures the higher economic efficiency. However, the effect of additional C to the T_100_ is limited to ΔT_100_ ≈ −105 °C. The vertically separated three data points in Fig. 4c, (0, 8), (2, 8), and (3, 8), show that the effect of C on the T_100_ increase as a function of C addition. Because C released from alkanes aggressively attack H_2_O and liberate hydrogens of H_2_O, the presence of excess C may increase the chemical potential and the reducing potential of gas phase H_2_^21^.

### Reaction mechanism of alkane reduction of SnO_2_

**Figure 5:**
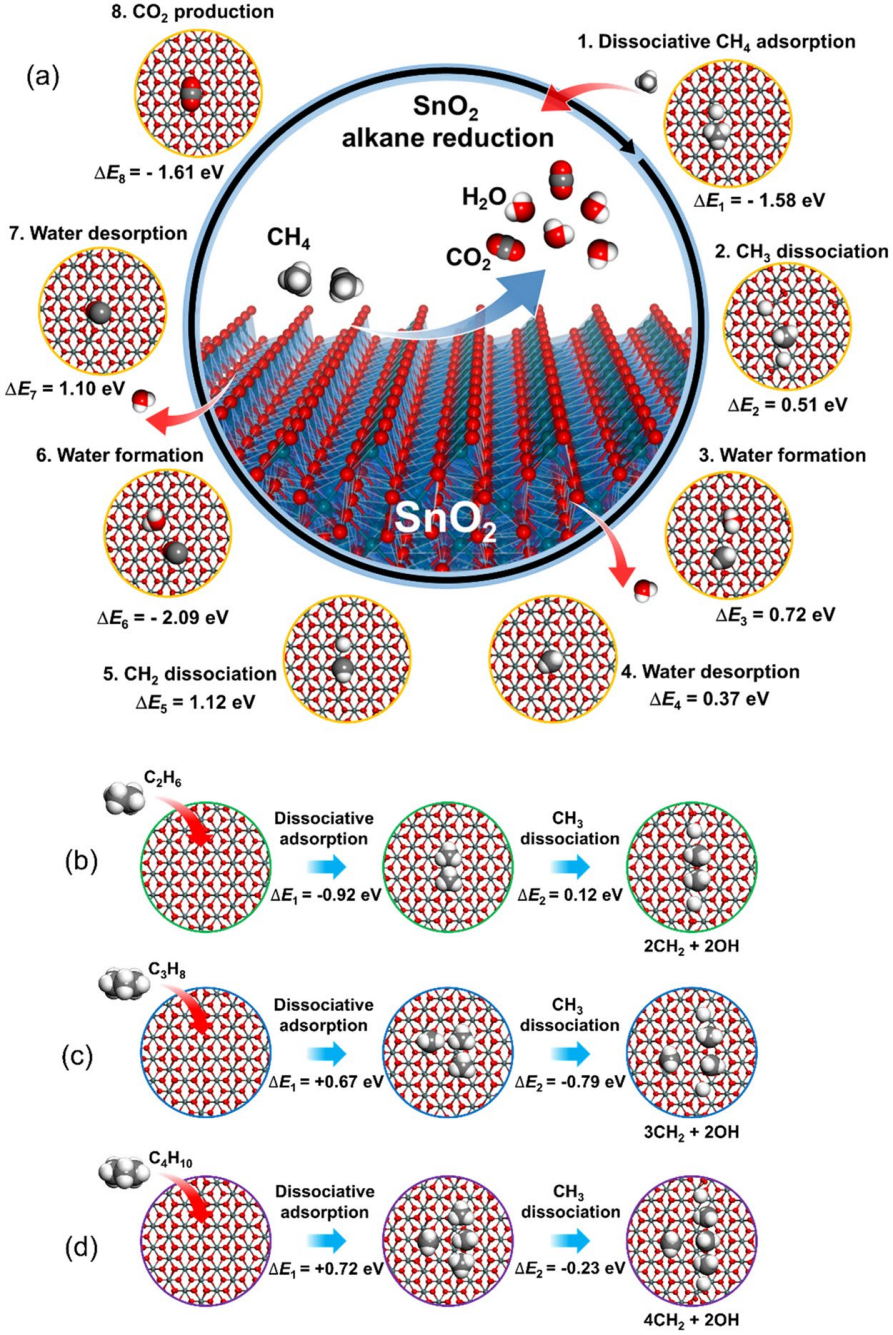
DFT-estimated mechanism of alkane reduction of SnO_2_. (**a**) CH_4_ was introduced as a prototypical alkane molecule to explore the atomistic mechanism of alkane reduction of SnO_2_. The reduction reaction proceeds clockwise following the numerical order. Δ*E*_n_ presents the energetics of the *n*^th^ process. White and grey spheres denote hydrogen and carbon atoms, respectively. Sn and oxygen atoms are colored in deep green and red, respectively. Because of the protruded hydrogen atoms of a methyl group, –CH_3_, formed upon dissociative adsorption of CH_4_, initially participate in the reduction process, H_2_O is the initial product. The original data for the reaction pathway and the morphology of each reaction stage are adopted from our previous report (ref.^21^). Dissociative adsorption of (**b**) C_2_H_6_, (**c**) C_3_H_8_, and (**d**) C_4_H_10_ on SnO_2_(100). Dissociative adsorption of alkanes of 2 ≤ *x* ≤ 4 produces multiple –OH and –CH_3_ groups. Because each –CH_3_ group was eventually dissociated into –CH_2_ and –OH, the mechanism and the energetic of the subsequent reactions follow the case presented in panel (a).

As a prototypical example of alkane reduction of SnO_2_, DFT-calculated reaction mechanism of CH_4_ reduction of SnO_2_ is presented in Fig. 5a. The original DFT-calculated reaction energy values were adopted from our previous publication (Under Creative Commons Attribution 4.0 International License)^21^. The initial CH_4_ dissociative adsorption (Process #1, Fig. 5a) initiates the CH_4_ reduction of SnO_2_. Because a CH_4_ molecule was dissociated into a –CH_3_ methyl group and a hydrogen atom, which are independently bound to surface lattice oxygen atoms of SnO_2_, the SnO_2_ surface will be strongly hydrogenated upon exposure to CH_4_. The sequential combined processes of dehydrogenation of –CH_3_ to –CH (Processes #2 to #5) and water formation (process #3 and #4) are energetically uphill. This is because two hydrogen atoms produced upon dehydrogenation of single CH_4_ molecule were used for water formation. As we discussed above, under the CH_4_ rich reduction conditions, the surface oxygen ions of SnO_2_ will be eventually hydrogenated and thus the endothermic dehydrogenation of –CH_3_ and water formation will not hinder the overall reduction of SnO_2_. The second water formation (Process #6 and #7) and CO_2_ production (Process #8) are strongly thermodynamically preferred. The overall reduction of SnO_2_ by CH_4_ shows that the hydrogen atoms of CH_4_ participate in the reduction process first and the residual carbon atom finally reduces SnO_2_. This finding is consistent with the equilibrium concentration diagrams (Figs 2 and 3) showing that H_2_O always forms first to CO and CO_2_.

Interestingly, upon initial adsorption of C_2_H_4_, C_3_H_8_, and C_4_H_10_, multiple –OH and –CH_3_ groups were formed as a result of dissociative adsorption of alkanes (Fig. 5b–d). Later, each –CH_3_ group was eventually dissociated into –CH_2_ and –OH, therefore the subsequent –CH_2_ dissociation, water formation, and CO_2_ formation processes would saturate into the same processes presented in Fig. 5a. The overall reaction mechanism of SnO_2_ reduction by alkanes (C_*x*_H_*y*=2*x*+2_, 0 ≤ *x* ≤ 4), therefore, is identical to each other except for the detailed energetics of the initial dissociative binding step. Interestingly, the initial dissociative adsorption of C_3_H_8_ and C_4_H_10_ are energetically endothermic (Fig. 5c,d). However, considering that the alkane reduction would occur under the high alkane partial pressure conditions^20,21^, the highly negative entropic contribution to the Gibbs free energy of binding, −TΔ*S*, will definitely compensate the positive Δ*E* of dissociative adsorption (Δ*E*_1_ in Fig. 5c,d)^24,25^, making the Δ*G* of dissociative C_3_H_8_ and C_4_H_10_ binding negative (exothermic). The roughly calculated highly negative −TΔ*S*^0^ at standard state^26^ of propane (−0.83 eV) and butane (−0.95 eV) confirm that the ΔG values of dissociative alkane bindings (Δ*G* = Δ*E* − TΔ*S*) are negative. The DFT-calculated mechanism of alkane (C_*x*_H_*y*=2*x*+2_, 0 ≤ *x* ≤ 4) reduction of SnO_2_ shows that the overall reaction mechanism is consistent within the alkanes that we applied (C_*x*_H_*y*=2*x*+2_, 0 ≤ *x* ≤ 4) for SnO_2_ reduction, irrespective to *x* and *y*. This result confirms that the significantly accelerated reduction potential of alkanes upon increase in *nx* is due to the quantitatively excessive supply of reducing agents by alkanes with the higher *nx*. As we have noticed in the introducing part, the presence of the already reduced liquid Sn metal may assist the direct reduction of alkanes. If this process occurs, the overall reaction will proceed under the higher H_2_ partial pressure conditions (under the total pressure greater than 1 atm) with excessive solid state carbon supply. Results on the mechanism of SnO_2_ reduction by alkanes under the various conditions (partial pressure and carbon content) will be reported in due course.

## Conclusions

We study the mechanism of alkane reduction of SnO_2_ for efficient low-temperature recovery of Sn from SnO_2_ using combined study of thermodynamic simulations and DFT calculations. Through a comparative analysis of the reducing power of H_2_ and commercially available alkanes (C_*x*_H_y=2*x*+2_, 0 ≤ *x* ≤ 4) toward SnO_2_ reduction, we scaled the reducing potential of studied reductants with T_100_, the temperature at which SnO_2_ is completely converted to metallic Sn. The alkanes with the higher *nx* and *ny* quickly complete the reduction at low T_100_. Moreover, the positive effect of *nx* on the T_100_ was quite prominent in all studied cases of alkane reduction of SnO_2_. The T_100_ of the SnO_2_ reduction by alkanes (*n*·C_*x*_H_*y*_) was significantly decreased from the T_100_ of pure hydrogen with the same amount of hydrogen atoms (*n*·H_*y*_). The fitted exponential curves of T_100_ plotted as a function of *ny*, presents that the effect of C on the T_100_ being saturated to ΔT_100_ ≈ −105 °C.

The C and H atoms released from alkanes sequentially reduce SnO_2_ to Sn and eventually to metallic Sn. The initial stage of SnO_2_ reduction by alkane is identical to the H_2_ reduction of SnO_2_; H_2_ takes up oxygen from SnO_2_. However, in the presence of the released C from alkanes, H_2_ of H_2_O is released as a gas phase molecule as C takes up oxygen from H_2_O. Because the gas phase redistribution between H_2_O, H_2_, CO, and CO_2_, caused by solid C occurs at above the T_100_, the role of the solid C released from alkanes is likely to adjust the chemical potential of hydrogen of H_2_O and H_2_, accelerating the reduction of SnO_2_ by H_2_. The DFT-calculated atomic scale mechanism of alkane reduction of SnO_2_ confirmed that the overall reaction mechanism is consistent within applied alkanes (C_*x*_H_y=2*x*+2_, 0 ≤ *x* ≤ 4).

Our results show that the alkane reduction of SnO_2_ is an effective recovery method of metallic Sn from SnO_2_ or SnO containing industrial wastes or from Sn ores. The low T_100_ values of alkane reduction and the maximum ΔT_100_ of −105 °C suggest that the alkane reduction of SnO_2_ assures high economically efficiency with economic value added that is held by the co-produced H_2_ and carbons.

## Methods

### Thermodynamic simulation

Thermodynamic simulations were performed with the HSC 6.0 code (Outotec Research, www.hsc-chemistry.com). The relative thermodynamic stability of various Sn, C, O, and H containing chemical compounds was estimated at temperatures between 0 °C and 1,200 °C. The initial equilibrium simulations were performed with 1 kmol of SnO_2_ balanced with increasing amount of H_2_ or alkanes. The T_100_ of several commercially accessible alkanes (C_*x*_H_y=2*x*+2_, 0 ≤ *x* ≤ 4), methane (CH_4_), ethane (C_2_H_6_), propane (C_3_H_8_), and butane (C_4_H_10_), were measured and compared with that of pure H_2_ to estimate the effect of carbon addition on the reducing power of a gas phase reductant. To generalize the effect of carbon, the measured T_100_ values were fitted to exponential curves.

### Density functional theory calculation

We performed density functional theory calculations with the Vienna ab-initio simulation package (VASP)^27^ with the Perdew-Burke-Ernzerhof (PBE)^28^ exchange-correlation functional to study the reaction pathway and the corresponding energetics of alkane (C_*x*_H_y=2*x*+2_, 0 ≤ *x* ≤ 4) reduction of SnO_2_. The most bottom SnO_2_ triple layer was fixed during the optimization to ensure the structural robustness of the slab models. The interaction between the ionic cores and the valence electrons was described with the projector augmented-wave method^29^. The valance-electron wave functions were expanded in the plane-wave basis set up to the energy cutoff of 400 eV. The convergence criteria for the electronic structure and the atomic geometry were 10^−4^ eV and 0.03 eV/Å, respectively.

The datasets generated during and/or analyzed during the current study are available from the corresponding author on reasonable request.
